# Understanding Sleep Disturbance Due to Stressors: An Exploratory Mixed-Methods Study During the COVID-19 Pandemic

**DOI:** 10.3390/ijerph23080969

**Published:** 2026-07-27

**Authors:** Muhammad Aljukhadar

**Affiliations:** Olayan School of Business, American University of Beirut, Bliss Street, Beirut 1107 2020, Lebanon; muhammad.aljukhadar@aub.edu.lb; Tel.: +961-1-350000 (ext: 3930)

**Keywords:** sleep disturbance, wind-down routines, guided-meditation apps, grounded theory, pre-sleep behavior, sleep intervention programs, major stressors

## Abstract

Major societal stressors such as pandemics and economic crises can significantly disrupt individual well-being through altered sleep patterns. This study uses a mixed-methods approach to explore the underlying causes and consumption expressions of sleep disturbance during the COVID-19 lockdown. Based on qualitative responses from 59 participants, the findings reveal three core disturbance patterns: delayed sleep schedules, increased sleep duration, and sleep restriction. These shifts were associated with a combination of emotional arousal, cognitive overload, and disrupted routines. Two dominant types of pre-sleep thought processes—pressing and comforting—appear to influence sleep outcomes. Despite the availability of mindfulness and guided-meditation technologies, most participants did not adopt these tools, citing either skepticism or inertia. The proposed model highlights the interplay between personal traits, pre-sleep behavior, and stress responses in shaping sleep. The study offers implications for public health initiatives, suggesting that targeted and accessible interventions are needed to support sleep health during a major stressor.

## 1. Introduction

Sleep is a fundamental component of human health and psychological resilience, playing a critical role in cognitive function, emotional regulation, and overall well-being. During times of crisis, however, sleep is often one of the first domains to be disrupted. The COVID-19 pandemic, an unprecedented global stressor, not only challenged healthcare systems and economies but also profoundly affected individual routines, behaviors, and mental health. As people were forced to adapt to new constraints, such as lockdowns, isolation, and heightened uncertainty, many experienced changes in their sleep patterns—ranging from insomnia and fragmented sleep to oversleeping or delayed sleep phases.

While recent research has documented a general decline in sleep quality during the pandemic, less is known about the subjective experiences and thought processes that underlie these disruptions. Sleep disturbance is not a monolithic phenomenon; it reflects a dynamic interaction between environmental conditions, emotional states, and behavioral habits. Understanding these interactions is critical for developing effective public health responses and designing sustainable behavioral interventions that can mitigate the negative effects of prolonged stress.

This study aims to explore how individuals experienced and interpreted sleep disturbance during the COVID-19 lockdown, using a mixed-methods approach to uncover patterns in their narratives. The mixed-methods approach started with reviewing the literature to derive a theory-based model to sleep disturbance, then conducting a qualitative study to verify the model’s constructs, categories, and levels, and finally analyzing the quantitative data to explore the constructs’ associations.

By focusing on pre-sleep cognition, wind-down routines, and engagement with digital well-being tools, the study proposes a model that links stress responses to sleep disruption through both psychological and behavioral mechanisms. These insights contribute to broader discussions on health sustainability, psychological adaptation, and the design of effective interventions in times of major crisis.

## 2. Literature Review

### 2.1. The Pandemic as a Major Stressor Shaping Sleep

The pandemic’s impact on sleep is undisputed. To delineate the magnitude of sleep problems during the pandemic, Alimoradi et al. performed a meta-analysis on 177 studies from 39 countries [1]. They found that a fifth of the population have sleep problems, and the latter significantly associate with depression and anxiety. In another meta-analysis on 44 studies from 13 countries, Jahrami et al. found that almost a third of the population had sleep problems [2]. The pandemic—a major stressor affecting many—is an independent variable that shapes habits and behaviors. Individual sleep and habits, such as their consumption of pandemic news, alerts, and updates, as well as their wind-down routine before bed, can be viewed as dependent variables.

Focusing on sleep during the pandemic, Mandelkorn et al. classified sleep problems in three categories: insomnia (difficulty falling asleep and waking up during the night), circadian rhythm (sleeping later/earlier than usual and/or waking later/earlier than usual), and daytime dysfunction (complaining about feeling unrefreshed in the morning, needing to take a nap, or feeling tired during the day) [3]. Sleep disturbance comprises any sleep that deviates from the regular and necessary one, including sleeping more, less, and the difficulty to fall or stay asleep. Using an Indian sample of full-time professionals and university students, Majumdar et al. showed the pandemic’s impact on sleep, reflected by more day sleepiness, fatigue, napping, and depression [4]. Surveying Italians before the lockdown’s end, Gualano et al. showed that more than 40 percent had sleep disturbance (17.4% had moderate or high insomnia) [5].

Ornell et al., among others, argue that the pandemic—through its economic, social, and health outcomes—increased fear and anxiety in healthy individuals, which alleviate sleep quality and mental health [6]. Franceschini et al. conclude that the risk factors of sleep disturbance during the pandemic were elevated levels of anxiety and stress and a shift in sleep schedule [7]. The role of psychological factors is documented as well. Casagrande et al. argue that individuals fearing direct contact with COVID-19 patients and individuals uncertain about future infection were more likely to have sleep disturbance [8].

The relation between stress, anxiety, and sleep has been shown. Anxiety “is a universal emotion and it would at times be maladaptive not to experience it; it is a necessary part of the response of the organism to a stress” [9] p. 251. Surveying about 2000 Taiwanese people, Li et al. noted that 56% reported sleep disturbance, and 11% developed suicidal thoughts [10]. They found that worry about the pandemic, poor social interaction and support, and low physical health correlate with sleep disturbance. Surveying 1515 Italians, Gualano et al. showed that depression and work-related anxiety correlate with sleep disturbance [5]. Xiao et al. examined the relations between stress, anxiety, social capital, and sleep quality using SEM and correlation analysis on the input of 170 Chinese individuals who had to self-isolate for 14 days [11]. They found that anxiety and stress reduce sleep quality by alleviating social capital. Surveying 2291 Italian in late March 2020, Casagrande et al. showed the relations between distress, anxiety, posttraumatic stress, and sleep quality [8].

In conclusion, medical and health science scholars highlight the stress and anxiety resulting from the pandemic as the underlying mechanism to sleep disturbance. The theory, however, affords additional explanations. People, for instance, were exposed to divergent levels of stress during the pandemic, e.g., those away from their families and loved ones and those struggling with furlough or unemployment would experience more stress. Likewise, people who form part of the front-line workers and those who have a family member belonging to front-line workers would experience more stress. In other words, the level of stress an individual experiences is idiosyncratic, which might or might not shape sleep disturbance. Moreover, an individual’s traits and habits modulate the experienced stress, thus sleep disturbance. Studying stress’ impact on sleep quality for pregnant women, Li et al. found individual resilience to shape the stress–sleep quality relationship [12]. Resilience moderates the effect of loneliness on sleep disturbance during the lockdown [13]. In addition, the cross-sectional method used in the literature cannot provide a full understanding of sleep disturbance, and scholars are advised to use other methods and to consider individual heterogeneity. The stress a person experiences, traits such as resilience and ability to cope, habits, and behavior adjustment all modulate the pandemic’s influence on sleep disturbance.

### 2.2. The Pandemic as a Disruptive of Healthy Consumption and Sleep

The pandemic mitigated healthy behaviors. Scholars have linked unhealthy behaviors such as low-quality diet and excessive alcohol consumption to stress and sleep quality [14]. Tobacco, computer use, caffeine, and negative family environment relate to sleep deprivation [15]. When legislators imposed strict regulations on people to self-isolate and quarantine, people changed their behaviors and habits. They spent dramatically more time on the internet and behind their screens. Naturally social, humans have an innate desire to interact and meet others. Indeed, virtual interactions and social media use increased voluminously during the lockdown.

Collecting sleep data through wearables from twenty countries in a longitudinal study between January and July 2020, Ong et al. showed the drastic influence of the pandemic [16]. They noted that while the pandemic has generally caused sleep disturbance, more disturbance happened in countries with stricter lockdown. They reasoned that mobility restriction (less physical activity) and self-isolation had dire effects on sleep. Surveying 1491 Australians in April 2020, Stanton et al. showed the unfortunate deviations in physical activity, sleep, and alcohol and cigarette consumption [17]. They noted that the deviations were amplified for individuals with high levels of depression, anxiety, and stress.

There has been a disruptive impact due to schools and universities closing their doors. Lectures and assignments moved to such platforms as Google Classroom, Microsoft Teams, and Zoom. Because league games and events were canceled, individuals played more video games [18]. E-sport streaming platforms saw record numbers. The use of social media apps such as Facebook, Instagram, and WhatsApp similarly shot up. People spent more time on digital activities, including social media and gaming, in countries with strict lockdowns; they adopted and used more e-commerce apps as well [18]. While social media apps offered convenient ways for people to connect, socialize, and have fun during the pandemic, their impact on sleep disturbance is not fully understood.

Ingram et al. surveyed 399 Scottish individuals during the lockdown and found that poor diet, sleep quality, physical activity, and negative mood correlate [19]. Sañudo et al. gathered objective data of physical activity, smartphone use, and sleep from 20 participants in Spain prior to and after the lockdown [20]. They found that during the lockdown, the participants exerted less physical effort, used their smartphone more, and slept more. Leone et al. also found that people slept longer and later during the lockdown, warning that their sleep schedule had shifted (chronotype has delayed), which disrupts the circadian rhythms and mitigates health and well-being [21].

The pandemic’s impact on sleep is not straightforward. Cellini et al. paradoxically found that while people slept more during the lockdown, they report lower sleep quality [22]. Blume et al. delineated the complex relation between lockdown and sleep [23]. Performing a quasi-experiment on Europeans from Austria, Germany, and Switzerland, these researchers argued that the lockdown increased the match between social and biological timing (less social jetlag)—a positive outcome that translates to more sleep time. They concurrently noted that the burden produced by the pandemic reduced sleep quality. Using wearable data, Ong et al. concluded that the lockdown rigorously reduced physical activity, and half of the sample (made up of younger and single people) showed the lowest physical activity [16]. However, these researchers could not observe an impact of the lockdown on objective sleep quality.

## 3. Theory Building: A Model of Sleep Disturbance During a Major Stressor

**Figure 1:**
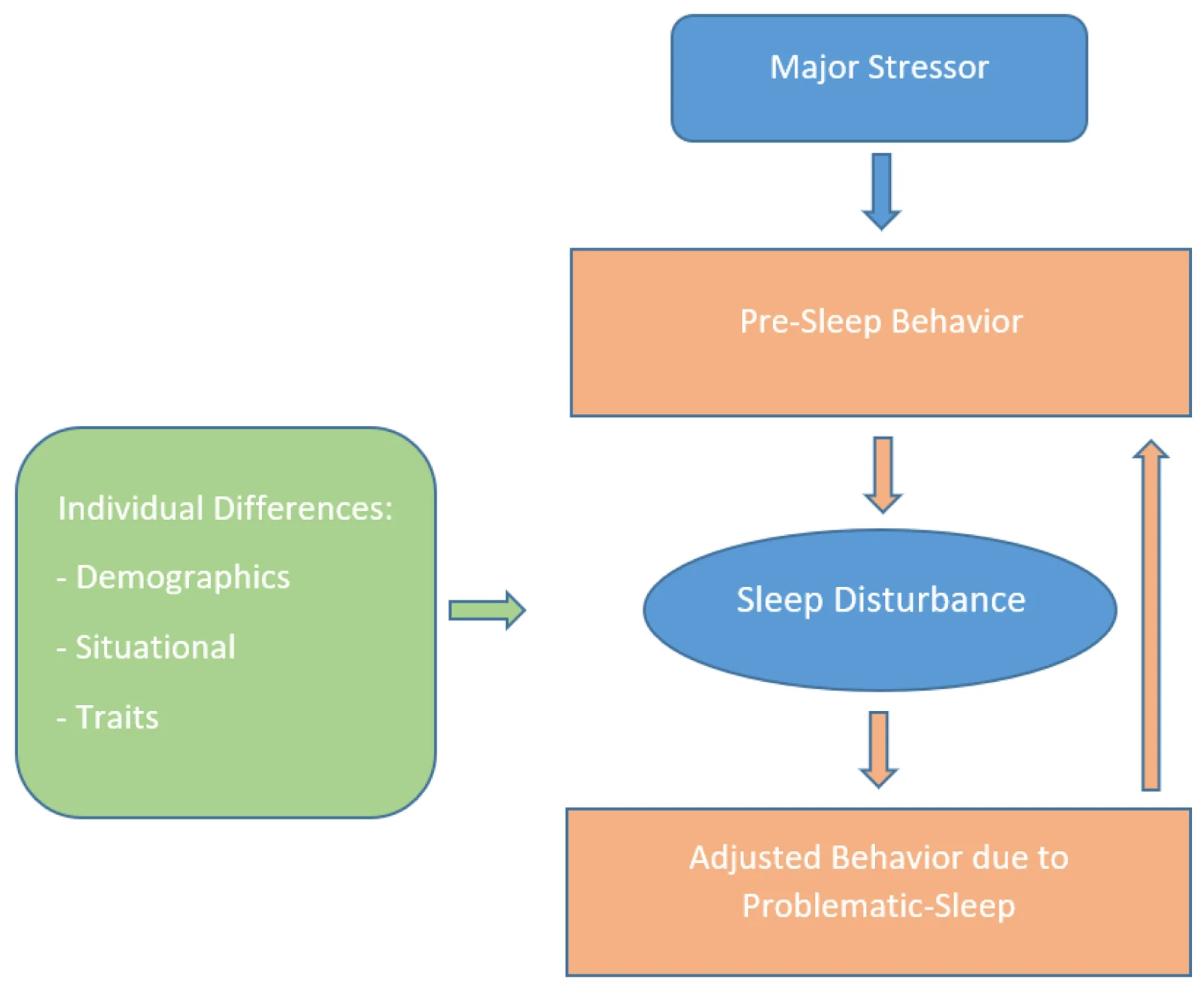
A general model of individual’s sleep disturbance during a major stressor.

We contend that the pandemic’s impact on sleep is complex—shaped by attitudinal, behavioral, and individual factors. A model of sleep disturbance should consider (1) how the individual’s pre-sleep habits and behaviors modulate the influence of a major stressor on sleep disturbance, (2) how individual differences (demographics such as age, gender, and occupation; situations such as the stress experienced and high-risk group membership; and traits such as resilience and ability to cope) modulate sleep disturbance, and (3) how the individual adjusts their attitudes, habits, and behaviors due to sleep disturbance. Such adjustments are vital because they delineate an individual’s copying strategy and because the new habits gradually replace the old ones, helping ascertain the long-term effects of a major stressor on sleep. The model’s generic constructs are depicted in Figure 1. The theory implies that pre-sleep attitudes and behaviors (e.g., wind-down routine, pandemic news consumption, and pre-sleep thoughts) modulate the impact of a major stressor on sleep.

### 3.1. Wind-Down Routine

An individual usually follows a wind-down routine before sleep to calm down and prepare. Highlighting the dearth of research on these routines, Koketsu [24] concluded that pre-sleep routines are underexplored in normal adult sleepers. Based on meta-analysis, Bartel et al. found that good sleep hygiene (e.g., using the bed only for sleep) and physical activity are associated with less sleep problems, whereas smartphone, gaming, and online activities are associated with late sleep [15]. Discerning an individual’s wind-down routine is crucial because consistency and automaticity characterize it.

Certain wind-down routines, such as smartphone use, affect sleep by reducing the secretion of the sleep hormone (melatonin) and by inducing neurophysiologic arousals that heighten alertness. Using a smartphone or other light-emitting devices (exposing the eye to the blue wavelength) before sleep induces sleep disturbance [25]. Gringras et al. explained that smartphones and similar gadgets expose the eye to short-wavelength light, especially when reading or playing games [26]. These researchers recommend wearing orange-tinted glasses or running specific apps to mitigate the penetration of this wavelength, thus improving sleep. In an experiment on 63 school students, Bartel et al. found that preventing smartphone use one hour before bedtime improves sleep [27].

Nonetheless, social media and screen time have a soothing pre-sleep effect. In fact, social media’s effect on sleep disturbance is disputable. Hale and Guan reviewed 67 articles studying the effect of screen time (e.g., TV, video games, and smartphones) on youth sleep [28]. They concluded that screen time is associated with negative outcomes, particularly sleep deprivation and a shift in sleep schedule. Nonetheless, they could not infer a causal relation (screen time → sleep disturbance) and highlighted several limitations in the articles they reviewed. Surveying 1310 Italians during the lockdown, Cellini et al. studied digital media use and changes in sleep patterns [22]. They found that while people spent more time on social media before sleep, this did not affect sleep. Social media and screen time might not be the cause of sleep disturbance. They might be a symptom of a sleep issue, or a symptom of a phenomenon shaping both. The pandemic, the anxiety, or the fear of missing out could shape both sleep disturbance and screen time.

Following the same reasoning, watching (Netflix, TV, YouTube, etc.) might be a soothing wind-down routine. The evidence suggests that watching when performed within limits has a positive impact on sleep. Surveying 423 youths, Exelmans and Van den Bulck found that reasonable watching does not mitigate sleep quality: only binge watching relates to poor sleep quality and fatigue [29].

An individual’s wind-down routine is largely stable, automatic, idiosyncratic, and abide to certain rituals. Studying 16 adults using projective techniques, Koketsu and Pierce indicated that normal sleepers described pre-sleep routines as occurring in certain locations, following similar sensory conditions, automatically, in a private manner and expectable, regular order [24]. The impact of social media and other wind-down activities such as watching and gaming on sleep disturbance is not clear. There is a need to categorize individuals’ wind-down routines during the lockdown then explore their impact on sleep disturbance.

### 3.2. Consumption of Pandemic News

After the outbreak, people turned to news portals but also to social media for updates and news on the pandemic. Surveying 7236 Chinese individuals during the pandemic, Huang and Zhao studied the impact of anxiety and depression on sleep quality [30]. They found that the time people spend focusing on the pandemic and its news associates with anxiety. An individual’s news consumption during a major stressor is thus a relevant factor. Such consumption might gauge stress and thus shapes sleep disturbance.

### 3.3. Pre-Sleep Thoughts

Strikingly, the attitudinal aspect is overlooked in sleep research. How people respond to a major stressor and the type of preoccupying thoughts before sleep should shape sleep disturbance. Surveying 1662 individuals, Grey et al. found that loneliness strongly shapes sleep disturbance for worried individuals [31]. Attitude toward sleep-promoting behavior affects pre-sleep behavior and thus sleep disturbance [32]. In addition, attitude toward sleep fosters pre-sleep thoughts and behavior. Individuals aware of the habits that promote sleep quality experience fewer sleep problems [33]. Cognitive interference (having worrying thoughts) before sleep is a characteristic of unhealthy sleep hygiene. Thoughts, whether negative or positive, should shape sleep disturbance.

### 3.4. Individual Differences

Scholars have focused on how demographics shape sleep disturbance. Other individual differences—be it situational or traits—were far less studied. Differences between groups shape sleep attitude, routine, adjustment, and, thus, disturbance. Mandelkorn et al. indicated that after the pandemic, forty percent of the population reported sleep problems [3]. They found that sleep disturbance was more pronounced for females: women suffered from sleep problems with eighty percent higher odds. Interestingly, about a fifth of the population reported improved sleep during the pandemic. Altena et al. elaborated that confinement has a good influence on sleep for some groups, including night owls and adolescents [34]. They argued that, by gaining more time, exposing themselves to daylight, and exercising, some individuals will have better sleep quality. In addition, some will have reduced stress resulting from remote work, which also improves sleep.

As for demographics, females and youths would have more sleep disturbance during a major stressor. Females, single people, and people between 18 and 45 years were at higher risk of having sleep and physical activity issues during the lockdown [17]. Franceschini et al. showed that a risk factor of having sleep disturbance during the pandemic was being female [7]. Casagrande et al. indicated that the youth and females were at higher risk of sleep disturbance during the pandemic [8]. Gualano et al. found that being female, younger, and living with no cohabitant during the lockdown associated with more sleep disturbance [5]. Huang and Zhao (2020) found that individuals aged less than 35 had higher anxiety during the pandemic [30]. Surveying 1242 Chinese people, Fu et al. studied the relations between anxiety, depression, copying style, and sleep disturbance [35]. They found that being female, having a lower income, and reporting low physical activity were risk factors for sleep disturbance.

Remarkably, the type of sleep disturbance appears to differ according to demographics. While many individuals suffered from sleep disturbance during the lockdown, the disturbance type differs by age group. Surveying 400 university students and staff, Marelli et al. found that during the lockdown, there was an increase in sleep disturbance [36]. They noted that students were affected by a shift in sleep schedule, whereas older staff were affected by difficulties sleeping and insomnia. Studying 139 university students before and during the lockdown, Wright et al. found that the students, while they had a shift in sleep schedule, had more sleep time during the lockdown and lower social jetlag (the difference in sleep time between regular days and the weekend) [37].

The effect of demographics on sleep disturbance is inconsistent though. Fu et al., for instance, suggest a peculiar impact of age: having a university degree or higher (not being a student) is associated with sleep disturbance [35]. Their results indicate that, contrary to other studies, the level of education/age positively relates to sleep disturbance.

### 3.5. Experiencing Nightmares

In addition to memories and flashbacks, nightmares are a form of re-experiencing a traumatic event [9]. The pandemic has increased the experience of nightmares. Surveying 419 Americans, Kennedy et al. found that individuals having more stress due to the pandemic experienced related nightmares [38]. Studying almost 6000 Italians during the lockdown, Scarpelli et al. found that sleep disturbance predicts nightmare prevalence [39]. They noted that the prevalence was higher for females, youths, and individuals with anxiety and depression. Surveying 1057 Brazilians, Musse et al. studied nightmares, documenting a dramatic increase in their prevalence during the pandemic [40]. They showed that nightmare experience increased for females, youths, and led to more alcohol consumption, more fear from contracting the novel coronavirus, and sleep medication. Including nightmare experience in the model is relevant because it gauges sleep quality. In effect, “… complaints such as insomnia or nightmares have even been incorporated in some anxiety disorder definitions, such as generalized anxiety disorder and posttraumatic stress disorder” [9] p. 249. Nightmares or bad dreams, being a form and outcome of sleep disturbance, should affect and be affected by pre-sleep behavior and adjustments.

### 3.6. Using Guided-Meditation and Relaxation Apps

When facing increased stress, individuals lean on strategies to manage it. A potential strategy comprises the adoption of helpful technology. Guided-meditation and relaxation apps (termed guided-meditation apps herein), such as the Calm app, help control stress and improve self-compassion [41]. These apps commonly train individuals to achieve relaxation and meditate. They build on the notion that paying attention to the present moment and identifying persistent thoughts and feelings help reduce stress and improve mental well-being. Cincotta et al. showed the potential of mindfulness and relaxation apps in alleviating sleep disturbance [42]. Highlighting the efficacy of these apps, Daudén Roquet and Sas indicated that a minority of them provide mindfulness training while the majority provide time reminders for relaxing and meditating [43]. In a randomized controlled experiment, Huberty et al. showed the efficacy of the Calm app in addressing sleep disturbance because the app reduces pre-sleep arousal, fatigue, and daytime sleepiness [41].

Given their efficacy, it might be in the interest of individuals to be aware of and use these apps during a major stressor. It is hence relevant to address individuals’ resistance toward the adoption of these apps during a major stressor. Top meditation and mindfulness apps were “Calm2”, “Health & Fitness App”, “Aura”, and “Headspace”. It should be noted that these apps are different from sleep monitoring apps, which come with wearables and are useful for some groups, i.e., individuals with sleep preconditions and individuals suffering chronic insomnia [44].

## 4. Qualitative Study

After advancing the theory-based model (Figure 1), we deduced the constructs in each theoretical block and their levels using a qualitative study. The survey’s nine questions were open-ended. This method, which is unstructured, enabled the collection of rich data on sleep-related attitudes and behaviors from more individuals during the COVID-19 lockdown. As instructed by our university ethics board, to minimize the chance of contracting and spreading COVID-19, the participants received the open-ended survey online. Participants were first provided with an introduction to the study, then asked to answer, in their own words, the following nine questions: (1) Have you been watching the news (related to COVID-19) and staying updated? (2) What is your current routine to wind down before bed? (3) Have you noticed any changes in your sleeping pattern since the start of the pandemic? If yes, in what way? (4) Are you experiencing a loss of sleep during the pandemic breakout? If yes, what is keeping you up? (5) Do you have any difficulties falling asleep, bad dreams, or difficulties staying asleep? (6) When I cannot sleep I… (7) What do you think about before you fall asleep? (8) Are you using any type of guided-meditation and relaxation apps like Headspace and Insight Timer? Why or why not? (9) Tell us a little about yourself (age, gender, income, and profession).

Fifty-nine individuals belonging to a convenience sample residing in Beirut, Lebanon and aged between 17 and 65 years (mean = 22.1 year, SD = 8.55) adequately responded to the open-ended survey during the last week of April 2020. According to gender, 59.3% were females, 37.3% were males, and 3.4% were other or unidentified. According to occupation, 42.4% were pre-university students, 35.6% were university students, and 20.3% were either graduate students, working, or unemployed. That is, the sample comprised more females and younger respondents. The majority (79.3%) were high school, college, or university students.

## 5. Coding and Results

**Figure 2:**
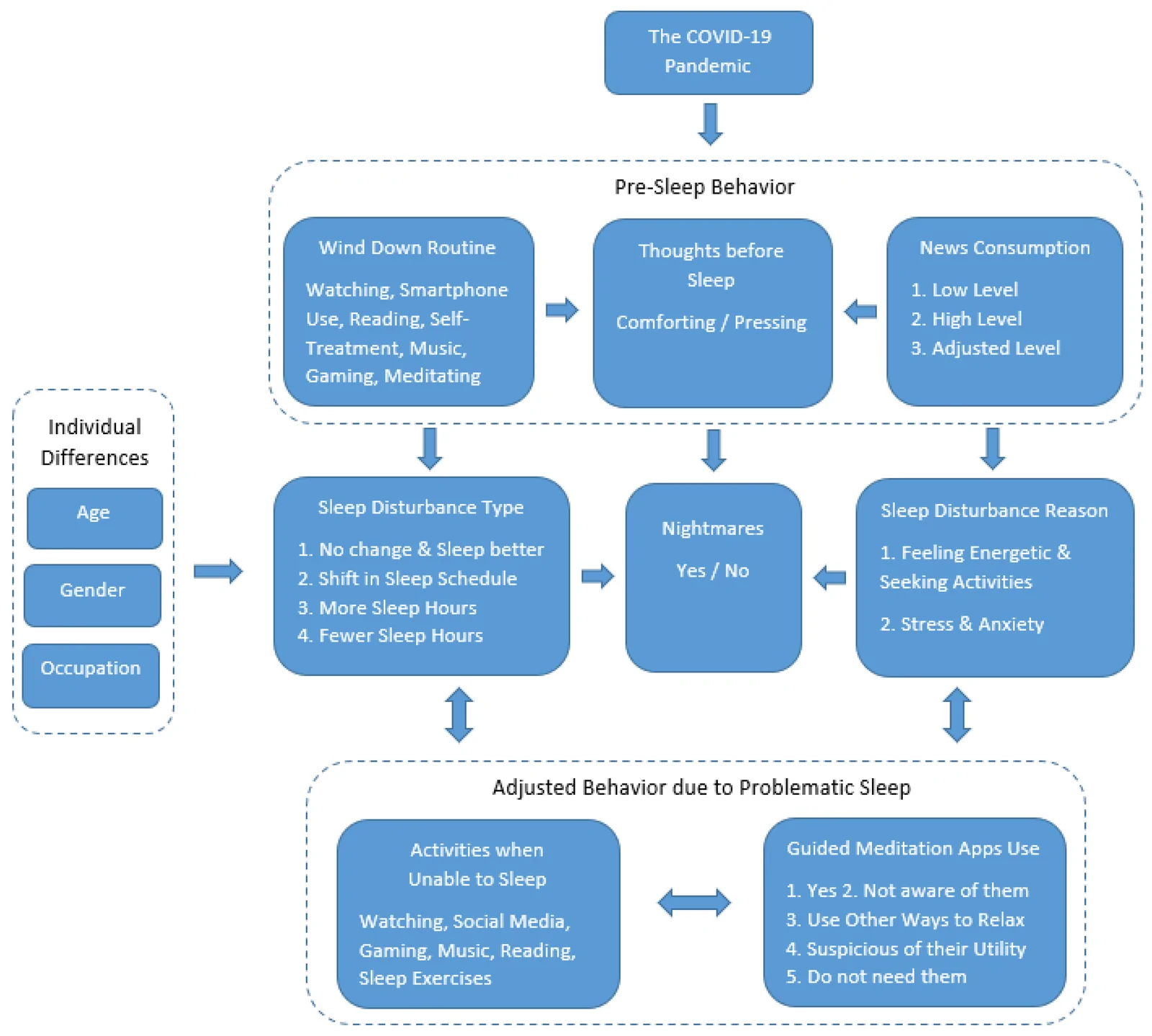
Resulting model of sleep disturbance during a major stressor.

Two research assistants coded the responses, resolving the disagreement between them. Then, the author reviewed the coding and applied necessary corrections. Recurring themes emerged for each construct, enabling the identification of meaningful levels. The responses for each question were coded into categories and levels; the details of the corroboration of model constructs and levels (summarized in Figure 2) are reported next.

### 5.1. Pandemic News Consumption

Consumption of pandemic-related news comprised three levels (Table A1 in Appendix A for coding and levels). The first, corresponding to a low level, comprised participants who did not stay updated or somewhat updated (22% of sample). The second, corresponding to a high level, comprised participants who stayed updated (66%). The third, corresponding to an adjusted level, comprised a third group (12%) that indicated they stayed updated at the beginning of pandemic but adjusted later by reducing their news consumption.

### 5.2. Wind-Down Routines

Eight activities embodied the wind-down routine (Table A2 in Appendix A). The participants reported a host of pre-sleep activities comprising reading (15.3%), using a smartphone (33.9%), gaming (6.8%), listening to music (8.5%), a workout (3.4%), watching Netflix, YouTube, or TV (50.8%), self-treatment (11.9%), and meditating (5.1%). We considered using a smartphone and watching as different activities (although watching can be performed using smartphone) because they involve different participation and interactivity levels. The most frequented wind-down routine before sleep was watching, followed by using smartphone, reading, self-treatment, listening to music, and gaming. Meditating and working out were the least frequent routines. To investigate the associations between the routines, we performed Cramer’s V, given that the variables are categorical. The analysis showed that most variables had little to no association (V = 0), suggesting that participation in one activity does not strongly predict participation in another for most categories. The analysis showed that the strongest, though modest, association was between self-treatment and meditating (V = 0.241). There was also a weak association between watching Netflix, YouTube, or TV with each self-treatment (V = 0.173) and using a smartphone (V = 0.140).

### 5.3. Types of Sleep Disturbance During the Pandemic

Four categories emerged (Table A3 in Appendix A). The first category (25.4% of the sample) reported no change to sleep or better sleep during the lockdown. The second category (45.8%) reported a shift in sleep schedule. The third (13.6%) reported more sleep hours. The fourth (15.3%) reported fewer sleep hours. We coded the levels as such, from one to four, to create a variable gauging the severity of sleep disturbance (mean = 2.19, SD = 0.99). The largest category was the one reporting a shift in sleep schedule during the lockdown. Moreover, two categories (28.9%) reported either more or less sleep hours than before the pandemic. Consequently, three-quarters of the sample (74.7%) suffered from sleep disturbance. In this order, a shift in sleep schedule is classified as less severe than more sleep hours because the former represents a circadian misalignment rather than a physiological deficiency or chronic health indicator. According to ICSD-3, schedule shifts like delayed sleep phase often maintain adequate sleep duration, resulting in cognitive impairments that are less acute than those caused by actual sleep loss. Conversely, more sleep hours serve as a clinical marker for Hypersomnia or poor sleep quality; meta-analyses, such as that by Gallicchio and Kalesan, highlight the U-shaped sleep curve, which links long sleep duration to higher mortality risks and cardiovascular disease [45]. Therefore, while a shift primarily disrupts the timing of rest, excessive sleep hours signify deeper systemic health risks and higher long-term mortality, justifying its higher placement on the severity scale.

### 5.4. Sleep Disturbance Reason

One category (45.8%) declared that they do not suffer sleep disturbance (Table A4 in Appendix A). For the rest, two reasons that explain sleep disturbance emerged. One was feeling energetic and seeking activities prior to sleep (25.4%). The other was stress and anxiety (25.4). Two participants (3.4%) did not provide useful input.

### 5.5. Nightmares During the Pandemic

While the majority did not experience nightmares, a significant group (20%) experienced nightmares during the lockdown. The input is reported in Table A5 in Appendix A. We coded having no nightmares as 0 and experiencing nightmares as 1.

### 5.6. Activities Undertaken When Unable to Sleep

Six activities were undertaken when the participants are unable to sleep: (1) Watching Netflix, YouTube, or TV was performed by 66.1%, (2) social media use was performed by 59.3%, (3) gaming was performed by 13.6%, (4) reading was performed by 5.1%, (5) listening to music was performed by 10.2%, and (6) sleep exercises or other were performed by 5.1% of the sample. We examined whether these activities were related using Cramer’s V test and found that while most variables show no association (V = 0.000), there was a weak association between “Watching Netflix, YouTube, or TV” and “listening to music” (V = 0.115). The results suggest that choosing one activity when unable to sleep is largely independent of choosing another.

### 5.7. Resulting Pre-Sleep Thoughts

Two types of pre-sleep thoughts during the lockdown emerged (Table A6 in Appendix A). The first are comforting thoughts (33.3% of the sample). The second are pressing thoughts (66.7%). The topics of comforting thoughts comprised relationships, gratitude, and recalling good times. The topics of pressing thoughts comprised financial fears, future uncertainty, academic performance, and family safety in view of the threats imposed by the pandemic. We coded comforting thoughts as one and pressing thoughts as two.

### 5.8. Adoption of Guided-Meditation and Relaxation Apps

Few participants (5.1% of the sample) indicated that they use guided-meditation and relaxation apps. Four reasons for not adopting these apps during the lockdown emerged (Table A7 in Appendix A). To create a factor delineating the resistance to the adoption of these apps, we arranged the reasons in the following order. The first reason is not being well informed about these apps (16.9%). The second is the use of other ways to relax before sleep (18.6%). The third is being suspicious about the utility and effectiveness of these apps (22.0%). The fourth is being relaxed and not in need of such apps (20.3%). Ten participants (16.9% of the sample) did not provide useful input.

This factor, with one already being a user of these apps and five being relaxed and not in need of such apps (mean = 3.43, SD = 1.242), shows that only a minor segment of individuals was using these apps during the lockdown and the other segment ascribes the reasons behind not using them. It shows that the participants not using these apps were almost equally distributed in the four resisting segments (not well informed, using other relaxation methods, being suspicious, and already being relaxed). Those in the second segment (using other relaxation methods) mostly practiced yoga, listened to music, meditated on their own, or employed social and pet therapy to relax and fall asleep.

For the data of the 59 participants after coding, see Supplementary Materials.

## 6. Exploring the Constructs’ Relations

Analysis was performed on the coded data using SPSS Statistics V21.0 (coded data is reported in Supplementary Materials) to explore the relations in the sleep disturbance model. To explore the associations between the model’s constructs, summarized in Figure 2, bivariate correlations were conducted. Correlation analysis was performed on the sample (Table S1 for the resulting correlations). In addition, correlation analysis was performed on the participants reporting sleep disturbance (N = 44; Table S2 for the resulting correlation table). One-sided *p*-values are used given the assumed directionality in the model and the exploratory nature of the investigation [46].

The results lend preliminary support to the relations in the model (Figure 2) and revealed thought-provoking nuances. Firstly, a relation between pre-sleep behaviors (i.e., the individual’s wind-down routine) and adjusted behaviors (i.e., the activities the individual performs when unable to sleep) was existent. The results show that likewise activities were related (the correlation coefficient, i.e., Spearman’s rho and its *p*-value are reported): smartphone use was associated with social media (β = 0.374; *p* = 0.002), gaming was associated with gaming (β = 0.484; *p* = 0.000), music was associated with music (β = 0.502; *p* = 0.000), and watching was associated with watching (β = 0.370; *p* = 0.002). The correlations also suggest that individuals with watching as a wind-down routine were more probable to perform sleep exercises (β = 0.228; *p* = 0.042) and less probable to listen to music (β = −0.230; *p* = 0.040) when unable to sleep. Individual differences shaped the wind-down routines. Age (β = −0.335; *p* = 0.005) and occupation (β = −0.356; *p* = 0.003) correlated with less smartphone use. Occupation correlated with less music listening (β = −0.235; *p* = 0.038). Individual differences also shaped certain behavior adjustments for the individuals with sleep disturbance: Older persons (β = −0.316; *p* = 0.018) and females (β = −0.309; *p* = 0.023) were less probable to adjust by listening to music when unable to sleep.

Secondly, the dimensions reflecting sleep disturbance (i.e., sleep disturbance existence, severity, and reason) correlated with pre-sleep and adjusted behaviors. Individuals who watch when unable to sleep were at higher odds of having sleep disturbance (β = 0.322; *p* = 0.006). Individuals who listen to music when unable to sleep had more severe sleep disturbance (β = 0.254; *p* = 0.048). Individuals who suffered sleep disturbance because of stress and anxiety were more probable to use meditation (β = 0.254; *p* = 0.029) and self-treatment (β = 0.294; *p* = 0.049) as wind-down routines. Individual differences appear to impact sleep disturbance by means of gender. Females had more instances of sleep disturbance (β = 0.263; *p* = 0.024), attributing it to stress and anxiety (vs. feeling energetic and seeking activities, β = 0.255; *p* = 0.030). The existence of nightmares—which helps gauge sleep quality and disturbance—relates to several factors. Individuals experiencing nightmares more probably had sleep disturbance resulting from stress and anxiety (β = 0.271; *p* = 0.041). Further, those consuming more news related to the pandemic (β = 0.225; *p* = 0.043) and those who read when unable to sleep (β = 0.266; *p* = 0.021) were at higher odds of experiencing nightmares.

Thirdly, the type of thoughts before sleep (comforting vs. pressing) relates to several constructs. Individuals with sleep disturbance attributed to stress and anxiety (vs. attributed to feeling energetic and seeking activities) reported more pressing (vs. comforting) pre-sleep thoughts (β = 0.339; *p* = 0.016). For individual differences, age (β = 0.342; *p* = 0.013) and occupation (β = 0.404; *p* = 0.004) correlated with pressing (vs. comforting) pre-sleep thoughts for the individuals suffering from sleep disturbance. Certain wind-down routines—watching (β = −0.224; *p* = 0.047) and listening to music (β = −0.243; *p* = 0.034)—appear to help ward off sleep disturbance by inducing comforting pre-sleep thoughts.

Fourthly, individual resistance to the adoption of guided-meditation and relaxation apps relates to several constructs. The results for this factor, with one representing the lowest resistance (already a user) and five representing the highest resistance, were the following: (1) consumers with sleep disturbance resulting from stress and anxiety (β = −0.352; *p* = 0.021) and those with pressing thoughts (β = −0.328; *p* = 0.027) had lower resistance, (2) consumers with certain wind-down routines:, such as reading (β = −0.339; *p* = 0.009), self-treatment (β = −0.242; *p* = 0.047), and meditating (β = −0.346; *p* = 0.007), showed lower resistance, and (3) females (β = −0.287; *p* = 0.024) showed lower resistance. Individuals using sleep exercises when unable to sleep also showed lower resistance (β = −0.241; *p* = 0.047).

## 7. Discussion

The Center for Disease Control has called sleep deprivation a public health epidemic. This study offers a grounded understanding of how individuals experience sleep disturbance during a major societal stressor, with the COVID-19 lockdown as a case-in-point. The findings indicate that sleep disruption is shaped not only by anxiety and negative emotional states but also by overstimulation, altered routines, and individual cognitive patterns preceding sleep. This nuanced view expands the existing literature that links pandemic-related sleep issues exclusively to stress or mental health challenges. By identifying distinct disturbance profiles—shifted sleep, increased sleep, and restricted sleep—this study highlights the heterogeneity in how people react to prolonged uncertainty.

A key contribution lies in the identification of two cognitive orientations before sleep: pressing thoughts (e.g., worry, problem-solving, or rumination) and comforting thoughts (e.g., escapist or soothing mental content). These thought patterns serve as psychological mechanisms that mediate the relationship between stress and sleep quality. This finding extends cognitive models of insomnia, which suggest that pre-sleep arousal—whether emotional or cognitive—is a major factor in disturbed sleep. Franceschini et al. surveyed more than 6000 Italians during the lockdown, concluding that half of the population had sleep problems due to stress [7]. Our study highlighted that feeling energetic could be a culprit. Several participants reported disturbances not because of distress, but due to heightened energy and increased engagement in stimulating activities, indicating that even positive arousal can undermine sleep health. Visual and hearing interference can reduce pressing thoughts and unfavorable emotions. Pre-sleep artistic experience was shown to reduce pre-sleep negative emotions and cognitive arousal [47]. (For an illustrative video, see https://dl.acm.org/doi/abs/10.1145/3290605.3300804, accessed on 5 October 2025.)

Certain wind-down routines have a favorable impact on sleep. Watching and listening to music appear to enhance sleep by inducing comforting thoughts; however, performing these activities when the individual is unable to sleep has negative outcomes. When unable to sleep, watching Netflix, YouTube, or TV relates to instances of sleep disturbance, and listening to music relates to the severity of the sleep disturbance. Such findings expose the complexity of the impact of pre-sleep activities on sleep, implying an asymmetric downstream impact.

Despite the increased availability and visibility of guided meditation and sleep-aid apps, adoption remained low in this sample. This finding suggests that technological solutions alone may be insufficient without deeper behavioral engagement and awareness. Participants’ inertia or skepticism toward these tools point to the need for improved framing, access, and perhaps personalization of digital interventions. Behavioral sustainability requires not only the availability of well-being tools but also active uptake and integration into daily routines.

The findings elucidate the copying strategies of individuals in the face of a major stressor. Individuals suffering from sleep disturbance resulting from stress and anxiety were more probable to have self-treatment and meditating routines. Consumption of news related to the pandemic was high for many, increasing for individuals who use social media or read when unable to sleep. Noticing their elevated level of news consumption, a group of individuals reduced their news consumption during the lockdown. Such findings indicate that an individual’s pre-sleep behavior is malleable, dynamic, and idiosyncratic.

While scholars pinpoint the female gender and being younger as risk factors of sleep disturbance during the pandemic, they have not elaborated the reasons. The results highlight gender, age, and occupation as risk factors. The female gender is associated with sleep disturbance. Females were also at higher odds of having stress and anxiety (vs. feeling energetic and seeking activities) as the underlying reasons of sleep disturbance. Alternatively, the effects of age and occupation on sleep could be attributable to the attitudinal aspect—developing pressing pre-sleep thoughts. From a public health perspective, these results underscore the importance of tailoring interventions to specific types of sleep disturbance. Rather than adopting a one-size-fits-all model, strategies should address both emotional stress and cognitive–behavioral patterns. Programs aimed at sleep hygiene and resilience must incorporate flexible components that consider personality traits, media consumption, and the role of cognitive load before bedtime. Furthermore, demographic characteristics—such as age and living circumstances—should inform the design of targeted support mechanisms.

In the broader sustainability context, sleep health should be recognized as a key dimension of psychological resilience and human capital. Just as sustainable environments promote physical health, psychological environments—routines, information exposure, and self-regulatory practices—must be cultivated to support sleep as a restorative resource. The conceptual model emerging from this study contributes to that goal by linking environmental stressors, individual responses, and sleep outcomes within an integrated behavioral framework. The literature suggests that a potential reason for the prevalence of sleep disturbance relates to coping and self-regulation. A lack of self-control impairs the ability to engage in sleep-promoting behaviors [32]. Individuals who can delay gratification (i.e., have high self-control) have better sleep quality [48]. Certain individuals—those more sensitive to stress and worry—would be at a disadvantage in the face of a major stressor. High-frequency heart rate variability upon worry predicts sleep disturbance during a major stressor [49].

Public servants should benefit from the results. Self-regulation training, for instance, has the potential to alleviate sleep disturbance for some groups. Research should examine whether self-regulation or a relevant trait (stress sensitivity, resilience, and copying skills) explains the vulnerability of high-risk segments to sleep disturbance in the face of major stressors. Such research helps decide which intervention (e.g., self-control training, meditating, or attitudinal change) is apt for high-risk groups.

## 8. Limitations

This study offers valuable insights into sleep disturbance during a major stressor; however, several limitations should be acknowledged. First, the sample was primarily composed of young adults (mean age = 22.1), which may limit the generalizability of our findings to older populations who might experience and cope with stress and sleep disruption differently. Second, data collection relied on self-reported narratives through an online survey, which may introduce recall bias and limit the depth of insight typically attainable through interviews. Third, the cross-sectional nature of the study precludes causal conclusions or a full understanding of how sleep patterns may have evolved over the course of the pandemic. Fourth, cultural and socioeconomic variables were not deeply explored, yet these factors can significantly influence stress responses and sleep behaviors. Fifth, the small sample size and the use of one-sided *p*-values to explore the associations between the constructs should be considered when generalizing the findings. Although one-sided *p*-values were used in the analysis, it is important to note that the majority of the *p*-values, being small, were also significant under a two-sided test, indicating evidence of statistical significance in both directions (from the 32 *p*-values found to be significant, 18 were significant at the two-sided test, i.e., *p* < 0.0025). Finally, while the study proposed a theory-based model and explored some relations between the constructs, its generalizability remains to be shown in broader and more diverse populations under different types of stressors and using robust analysis.

## 9. Conclusions and Recommendations

This study offers a nuanced understanding of how major societal stressors, such as the COVID-19 pandemic, can disrupt sleep patterns through a range of psychological and behavioral mechanisms. Using a mixed-methods approach, the research identifies not only traditional stress-related pathways (e.g., anxiety and rumination) but also less commonly discussed drivers such as positive arousal and altered daily rhythms. Intervention programs that educate about proper wind-down techniques have shown efficacy [33]. Moreover, self-regulation training has the potential to alleviate sleep disturbance [50].

The emergence of distinct sleep disturbance patterns—shifted sleep schedules, oversleeping, and restricted sleep—suggests that individuals respond to crisis conditions in diverse and personalized ways. Younger individuals suffer mainly from a shift in sleep schedule or more sleep hours; older individuals suffer mainly from less sleep hours/insomnia.

A key insight from this research is the role of pre-sleep cognition, particularly the dichotomy between pressing and comforting thoughts, in shaping sleep outcomes. These findings call for more targeted and psychologically informed interventions that go beyond generic advice on sleep hygiene. Public health campaigns should differentiate between types of sleep disturbance and integrate strategies that address both emotional and cognitive pre-sleep activity.

Moreover, the underutilization of guided meditation and mindfulness technologies, despite their proven benefits, indicates a gap between availability and behavioral uptake. Future interventions should not only increase awareness of these tools but also embed them in culturally sensitive and habit-forming frameworks to encourage sustained use.

Sleep is an essential human activity, and minor sleep disturbances can have significant impacts on people’s health and well-being. To promote sleep health as a component of sustainable well-being, policymakers, educators, and mental health professionals must consider the broader ecosystem in which sleep occurs—one that includes media exposure, stress levels, daily routines, and access to psychological resources. Strengthening this ecosystem is essential to building resilience not only during crises but also in everyday life. The model advanced in this work offers a backdrop for these initiatives.

## Data Availability

Data is reported in Appendix A and the coded data is reported in Supplementary Materials Table S3.

## Supplementary Materials

The following supporting information can be downloaded at: https://www.mdpi.com/article/10.3390/ijerph23080969/s1, Table S1: Correlation between the variables for all sample (N = 59); Table S2: Correlation between the variables for participants suffering from sleep disturbance (N = 44); Table S3: Coded Data.

## Appendix A

**Table A1:** Coded levels of pandemic news consumption.

Emerging Category	Respondent Input
1. Low level (not staying updated or somewhat staying updated)	“No. I do not believe in such news.” “Not really.” “Not much.” “Somehow.” “Kind of.” “Yes, but not too much.” “Every now and then yes.” “Only through statistics and the internet, no news.” The rest of the answers herein are “No.”
2. High level (staying updated)	“Yes, it is important to stay updated to know what is going on and this actually makes people realize how serious this is and that one must take precautions.” “Staying updated through apps and family members.” “Yes, most of the time.” “Regularly.” “Almost yes.” The rest of the answers herein were “Yes.”
3. Adjusted level	“At first, I watched a lot of news but after 2 weeks I slept on it, got used to the situation.” “Yeah I have, I watch less news on TV now if I am comparing to the beginning of the pandemic. However, I followed the health authorities’ Instagram page in order to keep myself updated on new regulations and increase in number of cases.” “Yes of course, daily but not constantly as that was making me anxious.” “I was at the beginning, but I have not really been keeping up lately.” “I used to at the beginning, now not as much.” “I have occasionally been following the news surrounding the virus. However, I do not find myself constantly looking for updates on the conditions. Most of the information I gain is through word of mouth.”

**Table A2:** Coded wind-down routines before sleep.

1. Reading	“Either read a book or…” “Reading”, “Read a book”, “Read”, “Read a book”, “Reading a book”.
2. Using a Smartphone	“Surf through social media.” “I get in bed at around 12 and sit until around 2 on Instagram before I doze off.” “Twitch” “Using my phone.” “Use my phone”. “Phone” “Use my phone.” “Check my phone.” “Talk to friends”, “Talk to my boyfriend till I get sleepy”, “Usually I stay on my phone until I get tired”, “I either read off my phone, scroll through social media, or zoom with my boyfriend”, “Check news on my phone”, “social media”, “socialize with friends or scroll through my social media feeds.” “check my phone until I’m tired”, “look at funny memes on Instagram”, “Listen to a podcast”.
3. Gaming	“Ps4”, “I usually play an online game with my friends”, “gaming until I pass out”, “Playing cards”.
4. Listening to Music	“Music”, “… Then listen to music”, “Listen to music.” “Listen to calming music”, “I usually listen to music”.
5. Workout	“Working out”, “Working out”.
6. Watching Netflix, YouTube, or TV	“Watch some episodes of friends.” “Watch a movie”, “Watch Netflix”, “Watching series”, “Watching a movie or TV shows on Netflix or short episode on You-tube.” “YouTube”, “… I would watch a few videos before bed then I would sleep early”, “watching a movie”, “Netflix TV”, “watch videos”, “TV shows”.
7. Self-treatment	“Skin care”, “I would have a cup of anise drink to relax and sleep.” “Post-shower hair treatment, skin care, …” “Eat”, “Drink Nescafé”, “wash up”, “wash my face”, “Shower”.
8. Meditating	“I use a guided meditation app to help me relax and drift to sleep”, “a prayer”, “meditation”.

**Table A3:** Coded types of sleep disturbance during the pandemic.

1. No Change	“Nothing too new”, “No”, “Not really, it is the same especially with Ramadan”, “Not much, I graduated end of December, so not much difference given that I had nothing to do anyway”.
1. Sleep better	“Now, my sleeping pattern is very organized. I sleep early around 9–12 am and wake 6–10 am.” “Yes, I am sleeping better.” “Yes, I am feeling more energized”.
2. A Shift in Sleep Schedule	“I started sleeping at 4–5 am, while I used to sleep at 2 am max”, “Yes, I stay up way too late, wake up late, and still am tired.” “I’m sleeping and waking up pretty later than usual”, “Yes! Sleeping really late and waking up really late too”, “my sleeping schedule has changed dramatically, sleep at 5/6 am wake up at 12/1 pm.” “Yes, I have started sleeping at 6 am lol”, “Yea, I have been staying up all night and sleep during the day”, “Yes, sleeping and waking up late”, “It hasn’t been that affected because I still go to work 3 days a week but I could say I am sleeping a bit later than on normal days.” “YES, trouble falling asleep at night.” “Staying awake all night and sleeping all day”, “Yes. I am staying up all night and sleeping during the day”, “Yes. I am sleeping later than usual.” “Yeah, I am staying up more and I feel more tired.” “Yes. Sleeping late… waking late due to COVID-19” “Yes, I have been sleeping later most nights”, “Yes, it is harder to fall asleep and actually stay asleep. (bad quality sleep)”, “Yes, it became worse! I used to sleep generally but now it became (unfortunately) a routine to sleep between 3–4 and wake up at 11–12 the next day. This is probably because I wake up a bit before class where before I used to wake up 3 h early to freshen up get ready and go to uni, and staying up”, “Yes, I used to fall asleep and more importantly be sleepy by around 11 pm and wake up at 7 am and I loved it. Nowadays I’m unable to fall asleep before 4 am and I wake up at around 11:30 am. I’m not comfortable with my sleeping schedule as it is, but I feel helpless since I am just not tired enough to get sleepy.” “Yes, working from home is a challenge and I usually don’t feel motivated during the day as usually my days would be spent outdoors or in university therefore I feel more motivated to work at night and this keeps me up”.
3. More Sleep hours	“Yes, I have been sleeping a lot more than usual... probably a result of boredom and depression”, “Sleepier than usual”, “yes, sleeping more”, “Yes, sleeping for longer hours or sleeping whenever throughout the day”, “Yes, I have been sleeping a lot more.” “Yes, I am sleeping for longer hours”, “Yes, I’m sleeping for longer hours. I stay up late and wake up late. Before the pandemic, I had work so I would wake up at 9 and sleep at 12.” “Yes. Sleeping more than usual”.
4. Fewer Sleep hours	“Yes, I barely sleep”, “Yes, sleeping very few hours every night.” “Less sleeping hours, and late sleeping time”, “I have been sleeping much less < 5 h”, “yes. I’m staying up late and waking up early”, “Receiving much less sleep”, “Yes. I have been sleeping quite late and waking up early.” “I’ve had more sleepless nights since the start of the pandemic and also have been staying up more late. I also tend to feel tired but not sleepy.” “I am not able to sleep since I do not feel fatigue. That is because there isn’t much activity during the day. Online learning makes me lazier unlike school were u have to engage in some physical motion during the day.”

**Table A4:** Coded sleep disturbance reason.

1. No Major Sleep Disturbance	“No. I still have the same sleeping schedule, so I guess I am fine.” “No, I stay up only because I want to. I prefer the night.” “No, I am sleeping more”, “No, I’m used to anxiety”, “No, been sleeping more”, “No I am sleeping 8 h or if anything more”, “No but just messed up sleeping schedule”. The rest of the answers herein are “No”.
2. Feeling Energetic and Seeking Activities	“yes because of my high energy”, “lack of feeling tired or exhausted”, “My body is not physically tired, and my circadian rhythm has been disrupted”, “Yes, my lack of tiredness is keeping me up”, “Netflix, chatting with friends”, “Yes, food, tv, and video games”, “Studying”, “Watching shows, boredom”, “Prolonged working hours”.
3. Stress and Anxiety	“General school stress”, “Anxiety”, “Stress and worry”, “Anxiety over the situation we are in.” “Stress”, “Now I only sleep 6 h maximum because of University pressures.” “Yes, overthinking, nervousness, and stress.” “Unmet deadlines”, “Yes, sometimes just thoughts keep me up for longer hours.” “my thoughts”, “Yes, I’m staying up late thinking about what’s going on and how our lives have been affected by it and how life as we know it may never be the same again. Then waking up 3 or 4 h later feeling like I just slept all night.” “I think I am having a poorer sleep quality. My thoughts are keeping me up”, “Yes… My own thoughts”, “Yes, it’s probably the lack of needing to get up early these days, and overthinking (a lot).” “I find myself drowning in my thoughts and reflecting on the current situation and its severity.” “Yes, overthinking, nervousness, and stress.” “Yes definitely, overthinking”.

**Table A5:** Coded nightmares during the pandemic.

0. No Nightmares	“No difficulties. No bad dreams. My sleeping patterns are much better.” “Not really”, “Ever since the pandemic, I have been sleeping perfectly fine.” “I haven’t had a full 7/8 h of sleep; I usually wake up several times during my sleep.” Several participants answered “No”.
1. Nightmares	“Yes, bad dreams or more like weird dreams. Ever since the pandemic and lockdown, I haven’t had a full 7/8 h of sleep, I usually wake up several times during my sleep.” “I’ve been having some weird dreams but nothing specific to the pandemic itself”, “More dreams”, “Bad dreams and [difficulty] falling asleep”, “… some absurd dreams about gross or contaminated things”, “Nightmares many times during sleep”, “I do have bad dreams but occasionally.” “bad dreams yes, but not difficulties in falling asleep or staying asleep”, “having bad dreams all the time”, “I’m having difficulty sleeping and having bad dreams all the time, and I wake up often during my sleep”.

**Table A6:** Coded thoughts prior to sleep.

1. Comforting Thoughts	“How I am grateful in life”, “My current state of mind (if I’m happy, sad, annoyed, etc.)”, “I pray”, “Relationships”, “My girlfriend”, “Friends”, “My crush”, “I think back to comforting times, prior to the pandemic, surrounded by family and friends.” “Nothing”, “I do not have negative thought”, “My family”, “The future”, “My future”, “Education”, “Sex, helps me fall asleep”, “Where I want to be in life”, “Things that would calm me or make me happy in a way”, “I let my imagination run wild since I recently finished my academic obligations.”
2. Pressing Thoughts	“Not knowing if I will be able to find a job”, “Unemployment”, “Studies”, “How much work I have the next day and how much I miss my home country and need to get back.” “My grades”, “My studies”, “Studies and educational life”, “[I think about] everything” “Unpredictability of situation”, “If I will be able to continue studies abroad since I have received acceptance in universities abroad and Lebanon is way too expensive for education.” “I think about how to ensure a better life for myself”, “What my future is going to look like”, “Not knowing what the future holds”, “Unanswered questions regarding the next few months”, “Simply, what comes next for me”, “When will all this be over?” “How much more well the virus affect our daily life and the consequences that it well have on us after”, “Safety of older family members”, “Family safety”, “I exhaust myself to sleep, I usually think about my grades”, “Deep thoughts go through my head. I’m not really fine with taking about it.” “How crazy what’s happening is. The whole world literally stopped functioning.”

**Table A7:** Coded resistance to the adoption of guided-meditation and relaxation apps.

1. Yes I use these apps	“I used Oak app several times to fall asleep”, “Yes, I tend to keep a lot in, so meditating clears my mind and calms it from the constant torment.” “Yes, such apps help me relax and fall asleep very easily.”
2. No. I am not well informed about these apps	“No, because I haven’t heard of them or what they do.” “No, did not know they existed”, “No, I haven’t tried any yet but I think I might.” “No, I do not know what these are”, “No, not aware of these apps.” “No I never tried them (maybe I should though) since I never had the need to until recently.” “No because I didn’t know about them”, “No I never heard about them”, “Haven’t used any apps, though I tried yoga at the beginning but directly gave up. I basically have no energy or motivation to do anything, the whole pandemic and life drastically changing in a matter of days is mentally draining.” “No, I didn’t think of it.”
3. No. I use other ways to relax before sleep	“Listening to music while sleeping”, “I only drink a cup of anise. After a full day of working and studying non-stop I would need a warm drink to calm me and make me less restless.” “No. If I want to meditate, I will usually just do so on my own without any apps or even music.” “No, I do yoga classes on zoom”, “no, I do yoga, I’m self-taught”, “No, I personally find it hard to meditate, I often do play relaxing soft music if I really find hard time falling asleep”, “No because I have something that keeps me relaxed which is my dog and my family”, “Meditate without a guided meditation”.
4. No. I am suspicious of these apps’ effectiveness	“No, I don’t find them helpful”, “No [they are] useless”, “No, because I do not feel like they work”, “No, because I have my own sleeping techniques which I use.” “No, I’ve tried it and it didn’t work at all”, “No because I don’t believe they work.” “No because I don’t need to” “no [I do not use them because] meditation doesn’t typically help me”, “no I don’t think those work for me”.
5. No. I am relaxed and do not need such apps	“Never thought of using them. I usually sleep fine”, “Nope I’m relaxed”, “No. I don’t need them. I am usually a relaxed person”, “No, because I do not need them”, “I have not been using them. I don’t feel the need to use such apps to help me relax.” “I don’t need it.”
